# Alginate Microencapsulation as a Tool to Improve Biostimulant Activity Against Water Deficits

**DOI:** 10.3390/polym17121617

**Published:** 2025-06-10

**Authors:** David Jiménez-Arias, Sarai Morales-Sierra, Ana L. García-García, Antonio J. Herrera, Rayco Pérez Schmeller, Emma Suárez, Álvaro Santana-Mayor, Patrícia Silva, João Paulo Borges, Miguel Â. A. Pinheiro de Carvalho

**Affiliations:** 1 Departamento de Producción Vegetal en Zonas Tropicales y Subtropicales, Instituto Canario de Investigaciones Agrarias, Finca “Isamar”, Ctra. de El Boquerón s/n, Valle Guerra, 38270 La Laguna, Tenerife, Spain; 2 Agroquímica, ICIA, Unit Associated with CSIC by IPNA and EEZ, Ctra. de El Boquerón s/n, Valle Guerra, 38270 La Laguna, Tenerife, Spain; 3 ISOPlexis, Center for Sustainable Agriculture and Food Technology, University of Madeira, Campus Universitário da Penteada, 9020-105 Funchal, Portugal; 4 Grupo de Biología Vegetal Aplicada (GBVA), Departamento de Botánica, Ecología y Fisiología Vegetal, Facultad de Farmacia, Universidad de La Laguna, 38200 La Laguna, Tenerife, Spain; smorales@ull.edu.es; 5 Departamento de Química de Productos Naturales y Sintéticos Bioactivos, Instituto de Productos Naturales y Agrobiología (IPNA-CSIC), 38206 La Laguna, Tenerife, Spainajherrera@ipna.csic.es (A.J.H.); raycohps@ipna.csic.es (R.P.S.); 6 Servicio General de Apoyo a la Investigación, Edifico SEGAI, Universidad de La Laguna, Avenida Astrofísico Francisco Sánchez s/n, 38206 La Laguna, Tenerife, Spain; smesegai@ull.edu.es (E.S.); asantanm@ull.edu.es (Á.S.-M.); 7 CENIMAT|i3N, Department of Materials Science, School of Science and Technology, NOVA University Lisbon and CEMOP/UNINOVA, 2829-516 Caparica, Portugal; jpb@fct.unl.pt; 8 Centre for the Research and Technology of Agro-Environmental and Biological Sciences, CiTAB, Inov4Agro, University of Trás-os-Montes and Alto Douro, Quinta de Prados, 5000-801 Vila Real, Portugal; 9 Faculty of Life Sciences, University of Madeira, Campus Universitário da Penteada, 9020-105 Funchal, Portugal

**Keywords:** alginate microcapsules, biostimulants, water deficit stress

## Abstract

Climate change is reducing agricultural productivity through altered weather patterns and extreme events, potentially decreasing yields by 10–25%. Biostimulants like pyroglutamic acid can enhance plant tolerance to water stress, but their rapid degradation in the soil limits effectiveness. Encapsulation in alginate matrices promises to be a good solution, protecting the compound and enabling controlled release. This study reports, for the first time, that encapsulated pyroglutamic acid markedly enhances drought tolerance in tomato and maize plants. The encapsulation strategy reduces effective concentration by an order of magnitude while significantly improving water use efficiency, photo-synthetic performance, and overall stress resilience. These findings demonstrate that alginate-based encapsulation substantially increases biostimulant uptake and efficacy, providing a novel and efficient strategy to mitigate water stress in crops, with important implications for climate-resilient agriculture. Two encapsulation methods for generating the alginate microcapsules are compared: ionic gelation with Nisco^®^ system and the electrospray technique.

## 1. Introduction

Plant production worldwide is a major concern in the context of climate change. The connection between climate change and agriculture is well-established. Global warming impacts agriculture by altering average temperatures, rainfall patterns, and increasing the frequency of extreme weather events like heatwaves, floods, and droughts. These changes also lead to shifts in pests, diseases, atmospheric carbon dioxide levels, ozone concentrations, and the nutritional quality of food [1]. Indeed, the frequency of extreme weather events is becoming more common, with the potential to decrease agricultural productivity by 10–25%, and this impact is expected to worsen over the next 50 years [2]. For example, water scarcity is projected to be higher than in the past in 83–84% of global croplands, making it crucial to develop tools that enhance crop tolerance to water stress [3]. Biostimulant utilization is an effective way to generally improve plant tolerance against stress [4], and in some cases, it can increase crop production [5,6]. These compounds are widely recognized for their ability to enhance plant stress tolerance and improve crop yields, while being eco-friendly and safe for the environment. Biostimulants are a key enabler of the European Union ambitious environmental and agricultural goals. By improving sustainability, enhancing soil health, and supporting the circular economy, they help farmers not only comply with EU regulations but also contribute to a more resilient and sustainable future for European agriculture. In this context, they are considered a promising tool to support the objectives of the European Green Deal and the “Farm to Fork” strategy, aimed at building a fair, healthy, and environmentally friendly food system [7]. It is interesting to note that some compounds can trigger tolerance mechanisms, making them a promising source for new commercial products [8]. Moreover, some authors discuss the potential of these treatments to counteract the side effects of climate change in agriculture [9]. On this regard, biostimulants encompass a plethora of disciplines among them: chemistry, biochemistry, plant physiology, genetics, and agronomy, constituting an interesting field of research. Biostimulants are extensively reviewed in plant science research, highlighting how they are an eco-friendly solution to face biotic and abiotic stresses and to enhance food production, with deep studies into their modes of action. Among the various compounds that can be used to enhance drought tolerance, pyroglutamic acid stands out due to its proven effectiveness under field conditions in crops such as lettuce [5] and maize [6]. However, while a single application of this biostimulant is effective under controlled conditions, achieving the same results in the field requires weekly doses. Therefore, we believe that significant improvements are needed to reduce the frequency of application and enhance the production-to-cost ratio.

Pyroglutamic, as an amino acid, is easily degraded by soil microorganism [10] and, as in the case of more than 90% of agrochemicals, it can be lost to runoff during application, causing significant economic losses and environmental hazards [11]. One potential solution to this problem is encapsulation, which can help to protect agrochemicals and reduce runoff in agriculture. Encapsulating active ingredients offers several advantages, including a slower and more controlled release, greater efficiency, and enhanced safety for both users and the environment [12]. Although most encapsulation efforts in agriculture have focused on pesticides and fertilizers, research into the encapsulation of biostimulants is still limited, even though it holds significant promise for advancing agricultural practices [13].

Natural polymers like alginate offer promising possibilities for agricultural applications due to their low cost and compatibility with scalable techniques such as ionotropic gelation and with or without electrospray techniques [13]. The production of calcium alginate beads via ionic gelation is a simple and cost-effective method for encapsulating various materials. Calcium ions bind to the carboxyl groups of alginate, forming a gel structure [14]. Another notable advantage of using alginate carriers is their easy degradation under field conditions [15], making them an effective means of protecting the active compound without causing ecological harm. Alginate beads have been utilized to enhance the properties of biostimulants, particularly in microorganism-based formulations [16]. Encapsulation within alginate matrices aims to improve cell viability and can be used for various purposes, such as increasing plant tolerance to drought [17,18,19] or salinity [20]. However, at the time of writing this article, no research has been found on using alginate with other types of biostimulants, such as pure organic active compounds.

In this study, we explored for the first time the microencapsulation of pyroglutamic acid in calcium alginate beads, produced employing two different gelation methods: the electrospray technique and a Nisco^®^ system. The biostimulant activity against water deficit stress was evaluated for both microcapsules. The results demonstrate that the encapsulated pyroglutamic acid can significantly enhance the efficiency of the biostimulant, delivering the benefits of the amino acid to plants at doses ten times lower than usual. To the best of our knowledge, the application of encapsulation technologies in the development of biostimulant formulations remains largely unexplored. This study therefore provides a promising strategy to enhance the delivery and efficacy of amino acid-based biostimulants, offering a potential tool to mitigate crop losses associated with climate change-induced stress conditions.

## 2. Materials and Methods

### 2.1. Materials

Alginic acid sodium salt (Sigma-Aldrich, St. Louis, MO, USA), calcium chloride anhydrous (Sigma-Aldrich, St. Louis, MO, USA), and L-pyroglutamic acid (≥99% Sigma-Aldrich, St. Louis, MO, USA) were used. Ultrapure water was used throughout this study.

### 2.2. Preparation of Alginate Microparticles

Microparticles were produced via ionotropic gelation using 1% alginate as the polymer, containing 1 mM pyroglutamic acid and 5% calcium chloride as the gelling solution, at room temperature (25 °C) and 70% relative humidity. Two devices were employed for microbead production: a Coaxial Airflow-Induced Dripping VAR J1 system (Nisco^®^, Nisco Engineering AG, Wehntalerstr, Switzerland) and a custom-built electrospinning apparatus from the Biomaterials Lab at CENIMAT|i3N (Electrospray, Lisbon, Portugal).

The Nisco^®^ equipment operates on the principle of producing polymer beads in a controlled manner by utilizing a coaxial air stream to displace droplets from a needle tip into a calcium chloride gelling bath before gravity induces their fall. A polymer flow rate of 15 mL/h and an air flow rate of 5 L/min were used. The needle had a diameter of 0.15 mm, and the distance from the needle tip to the gelling bath was set at 10 cm (Supplementary Figure S1).

Electrospray, a technique derived from electrospinning, is a physical process in which polymer fibers are drawn by the action of an electric field without requiring additional mechanical energy. In this method, the polymer solution is contained in a syringe, with the needle acting as the anode and the cathode positioned at the bottom of a gelling bath where the particles are deposited. The parameters used in this experiment were a voltage of 8 kV, a syringe diameter of 1.9 mm, and a needle with a caliber of 27 µm. The distance between the syringe tip and the gelling bath was 16 cm, with the bath positioned 10 cm below the syringe (Supplementary Figure S2). To prevent particle aggregation, the gelling bath was placed on an electric stirrer.

### 2.3. Pyroglutamic Acid Determination

Analyses of pyroglutamic acids were carried out in a Waters Acquity UPLC^®^ I-Class, constituted of a sample manager with flow-through needle and a binary solvent. The UHPLC system was coupled to an MS Xevo^®^ G2-XS quadrupole time-of-flight (QToF) detector using an electrospray ionization interface in the positive mode. The MasslynxTM V4.1 software was used to control the pumps and sample manager, as well as MS parameters and process of spectrum data. Separation was carried out in bioZen Glycan column (100 mm × 2.1 mm, 2.6 µm) using a pre-column with the same characteristics (5 mm × 2.1 mm, 2.6 µm). The column and pre-column temperatures were settled at 40 °C. All instrumentation parts and software are from Waters Chromatography (Milford, MA, USA).

The mobile phase was composed of acetonitrile (solvent A) and water (solvent B), both containing 10 mM ammonium formate (pH 3.1 with formic acid). The composition was initially established at 100% A. It was changed to 95/5 (*v*/*v*) in 2.0 min and then to 50/50 (*v*/*v*) in 5 min and held for another minute. Finally, the initial conditions were set up in 0.1 min and maintained for 4.0 min until system stabilization. The flow rate was 0.5 mL/min and the injection volume was 5 µL at 10 °C.

The MS analysis was performed in the MSE Centroid mode using exact-mass precursor and product ion. Protonated ions, product ions (130.051, 84.045), and retention time were used as identification points, whereas a maximum tolerance of ±30% for the relative ion intensities of confirmation to quantification ions, with respect to the reference ion ratio, was established (2002/657/EC: Commission Decision of 12 August 2002 implementing Council Directive 96/23/EC concerning the performance of analytical methods and the interpretation of results). The source conditions were as follows: capillary voltage of 1.0 kV, source temperature of 120 °C, desolvation temperature of 500 °C, cone gas (N_2_) flow rate of 20 L/h, desolvation gas (N_2_) flow of 1000 L/h, and collision gas (Ar) pressure of 0.5 bar. The MS system was operated in the sensitivity mode in a *m*/*z* range of 50.00–600.00. For low energy fragmentation, the collision cell was set at 4 V, whereas for high energy, a collision energy ramp from 10 to 45 V was used. The cone voltage was set at 40 V. During the analysis time, a LockSpray correction was applied by acquiring Leucine enkephalin (556.2771 Da) for 0.5 s in an interval of 30 s. Chromatogram example is show in Supplementary Figure S3.

### 2.4. Microparticle Encapsulation Efficiency Determination

Using the detection protocol previously described, the encapsulation efficiency (EE%) was calculated using the following formula: (total drug added—free non-entrapped drug)/total drug added. Loading capacity refers to the amount of pyroglutamic acid loaded per unit weight of the microparticle, indicating the percentage of the microparticle’s mass attributable to the encapsulated drug. All determinations were performed in triplicate using 350 mg of microparticles to ensure that enough pyroglutamic acid was encapsulated to be detected using HPLC.

### 2.5. Microparticle Size Determination Using Scanning Electron Microscope

Nisco^®^ and electrospray microparticles were observed using a scanning electron microscope (Zeiss EVO15, Jena, Germany). The samples were mounted on metal stubs and coated with a 15 nm gold layer using a sputter coater (Quorum Q150RS plus, Quorum Technologies Ltd., Laughton, UK). Particle sizes were measured by analyzing 20 individual particles and determining their diameter using the free ImageJ software (Ver. 1.54P with help of the scale bar).

### 2.6. Plant Material and Experimental Conditions

Seedlings of *Solanum lycopersicum* L. and *Zea mays* L. were supplied by a local nursery vendor. Sowing was conducted in trays containing a commercial substrate using an automatic seeder to ensure uniform germination and growth until the two true leaf stage (BBCH-scale 12). The trays contained 150 cells, each measuring 3.5 cm in length and width, with a depth of 7 cm. Only well-rooted, disease-free seedlings of uniform size were selected for the experiments.

The seedling trays were maintained in a growth chamber under controlled conditions: temperature, 24 ± 2 °C; photoperiod, 16 h light/8 h dark; relative humidity, 60–75%; and irradiance, 300 μmol m^−2^ s^−1^.

### 2.7. Treatments and Water-Deficit and Rehydration Growth Assay

A water-deficit growth experiment was conducted over 7 days using tomato (*Solanum lycopersicum* L.) and maize (*Zea mays* L.) seedlings, following the procedure described by Jiménez-Arias et al. [21] Water stress was induced by irrigating with 50% less water using a half-strength Hoagland solution, compared to control plants irrigated daily at full field capacity. This irrigation was applied consistently to all drought-exposed plants.

A second experiment was conducted exclusively with tomato plants. This experiment involved an initial 7-day water-deficit period, followed by complete rehydration and an additional 7 days of growth.

In both experiments, plants were treated directly at the root zone with 5 mL of each treatment (Table 1) on the first day of the trial. Two hours later, well-watered plants received an additional 5 mL of a half-strength Hoagland solution. The treatments applied throughout the experiments are summarized in Table 1.

### 2.8. Growth Measures and Stress Index Calculations

In the first experiment, 10 seedlings from each treatment were collected after 7 days of drought. The second experiment was repeated with 20 plants per treatment, collecting samples after 7 days (water-deficit period) and 14 days (rehydration period). The plants were dried completely in an oven at 60 °C for 48 h and weighed individually. Using data from the second experiment, various indices were calculated, including the stress susceptibility index (SSI), stress tolerance index (TSI), relative growth rate (RGR), and plant water use efficiency (WUE_p_) (Table 2).

### 2.9. Gas Exchange Measurements

Gas exchange analyses were performed on fully developed leaves (N = 30). The photosynthesis rate (Pn), stomatal conductance (Gs), and transpiration rate (E) were measured on attached leaves using a portable infrared gas analyzer (LCPro, BioScientific Ltd., Hoddesdon, UK). Measurements were conducted under ambient CO_2_ concentration, a photosynthetic photon flux density (PPFD) of 1000 μmol m^−2^ s^−1^ (optimized with a light curve), and a cuvette airflow of 500 mL min^−1^. The values for instantaneous water use efficiency (iWUE) and intrinsic water use efficiency (intWUE) are the ratios between Pn/E and Pn/Gs, respectively [22]. The ratio between Pn and Ci was also calculated.

### 2.10. Pyroglutamic Acid Determination in Tomato Plants

Pyroglutamic acid determination in tomato plants was carried out as follows. Tomato leaves were harvested at 1, 3, and 7 days after the start of the experiment and immediately frozen using liquid nitrogen, collecting two replicates per treatment. The samples were ground, and 25 mg was used for extraction. The extraction process involved the addition of 1.5 mL of MeOH, vortex mixing, followed by sonication at 4 °C for 15 min, shaking for 30 min, and centrifuging for 10 min at 10,000 rpm. After centrifugation, the soluble fraction was transferred to a new microtube and dried (SpeedVac, Thermo Fisher Scientific, Waltham, MA, USA). The samples were then resuspended in 400 µL of µL of a mixture (aqueous Hammonium formate (10 mM)/acetonitrile 3:1) and filtered using a 0.22 µm filter. The filtrates were directly used for pyroglutamic acid determination by HPLC-MS/MS, as described in Section 2.3.

### 2.11. Statistical Analysis

One-way ANOVA tests (Duncan’s post hoc) were applied to analyze the differences between treatments in all measured variables, or a Student’s *t* test were used. All statistical analyses were performed using the IBM SPSS 24 statistical package.

## 3. Results

### 3.1. Pyroglutamic Acid Calcium-Bound Alginate Microbead Generation Study

**Figure 1:**
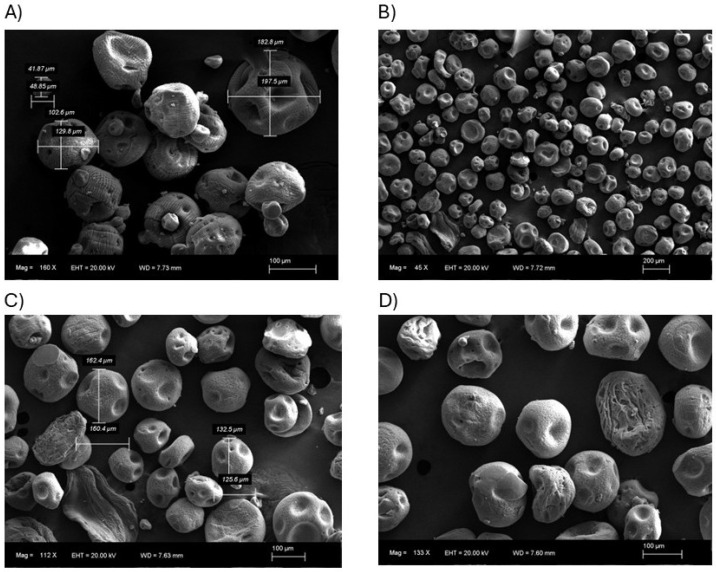
Microparticles shown using scanning electron microscopic imaging. Pyroglutamic loaded in calcium alginate beads generated by ionic gelation with Nisco^®^ equipment (**A**,**B**) and the electrospray technique (**C**,**D**).

Microalginate bound with calcium ions were created using two different approaches. The first one was generated by ionic gelation employing the coaxial airflow from Nisco^®^ (Nisco Engineering AG), yielding “Nisco^®^ microparticles”, and the second one was created using the electrospray technique, creating “electrospray microparticles”. Figure 1 shows the aspect of both particles using a scanning electron microscope.

**Figure 2:**
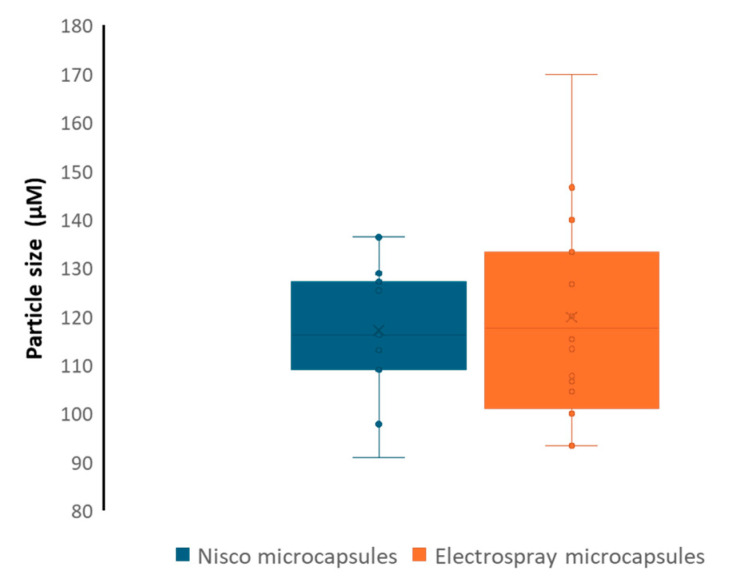
Size distribution of the Nisco^®^ and electrospray microparticles (N = 20 beads).

The average size of the particles produced using both techniques is approximately 120 µm, as shown in Figure 2. However, the Nisco^®^ microparticles demonstrate significantly less size dispersion, which is a noteworthy advantage when considering the consistency and homogeneity required for industrial-scale product manufacturing (Figure 2). This reduced variability in particle size enhances the potential for reproducibility and quality control in large-scale applications.

Our data suggest that both types of particles release their charge very quickly, specifically after 1 h in water with agitation or 3 h without agitation, exhibiting a similar profile. However, it is interesting to point out the difference in encapsulation efficiency, as the particles generated by the Nisco^®^ system reach on average 74.5% entrapment of pyroglutamic acid compared to those generated by the electrospray technique, which reach only 33.2% (Table 3).

### 3.2. Alginate Particles Loaded with Pyroglutamic Acid Are Capable of Inducing Tolerance with a Ten Times Lower Amino Acid Concentration

**Figure 3:**
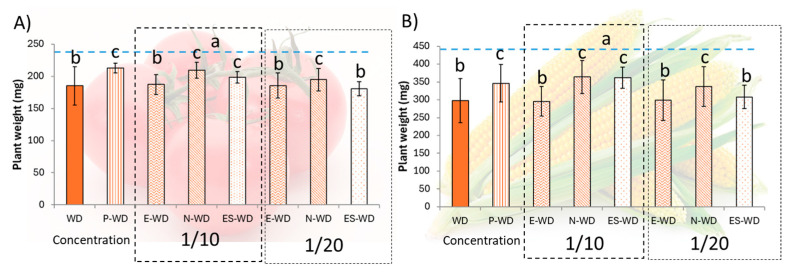
Average plant weight with standard deviation after 7 days of growth (N = 10 seedlings) in tomato (**A**) or in maize seedlings (**B**). Blue dashed line means well-watered plants’ average. WD means plants subjected to water deficit; P means pyroglutamic treatment; E means empty microparticles treatment; N means Nisco^®^ microparticles treatment; ES means electrospray microparticles treatment. Same letters show no significant differences (*p* < 0.05) using One-way ANOVA tests (Duncan’s post hoc).

To evaluate the impact of different treatments under water stress, a test was conducted in tomato and maize seedlings using a 50% water reduction (Figure 3A,B). The results demonstrate that pyroglutamic acid alone (P-WD) significantly increases tolerance to water deficits in tomato or maize plants. Empty particles (E-WD) showed no improvement in plant tolerance. However, the encapsulation effect is evident when Nisco^®^ (N-WD) and electrospray (ES-WD) microparticles were used under water-deficit (WD) plants. Both treatments provided enhanced stress tolerance to the plants despite employing ten times lower concentrations of pyroglutamic acid within the encapsulation process (1/10). Notably, N-WD increased tolerance even with 50% fewer particles.

A novel experiment utilizing only Nisco^®^ microparticles (using 1/10 dosage) in tomato plants was conducted, incorporating an initial period of water deficit (seven days) followed by complete rehydration. This approach revealed significant differences among treatments, particularly when analyzed through various growth and stress indices (Table 4).

During the water-deficit phase, untreated plants exhibited a pronounced reduction in the relative growth rate (RGR), with values decreasing by nearly 50%. In contrast, the application of pyroglutamic acid, both in its free form and encapsulated within microparticles, enhanced water-deficit tolerance by approximately 40%. Notably, water use efficiency (WUE) was slightly higher in plants treated with the encapsulated formulation compared to the free amino acid.

Under water-deficit conditions, plants treated with pyroglutamic acid displayed the lowest values in the stress susceptibility index (SSI), with the most pronounced improvement observed in those treated with pyroglutamic acid-loaded microparticles (N-WD). This outcome underscores the enhanced drought tolerance conferred by the encapsulated biostimulant.

Furthermore, the encapsulated pyroglutamic acid consistently achieved higher levels in the stress tolerance index (STI), highlighting its efficacy as a biostimulant to improve plant resilience under drought conditions. This evidence supports the use of pyroglutamic acid, particularly in its encapsulated form, as a promising strategy to mitigate the adverse effects of water stress in tomato cultivation.

However, after rehydration, differences under water-deficit conditions persist, although they are smaller than those observed after the first 7 days between pyroglutamic-treated plants (P-WD and N-WD) and untreated ones (WD) subjected to water deficit stress. Nonetheless, it is noteworthy that N-WD plants reach the lowest value in SSI index, showing an interesting behavior at the end of the experiment.

### 3.3. Alginate Particles Loaded with Pyroglutamic Acid Enhance Amino Acid Absorption and Improve Gas Exchange Parameters in Tomato Plants

**Figure 4:**
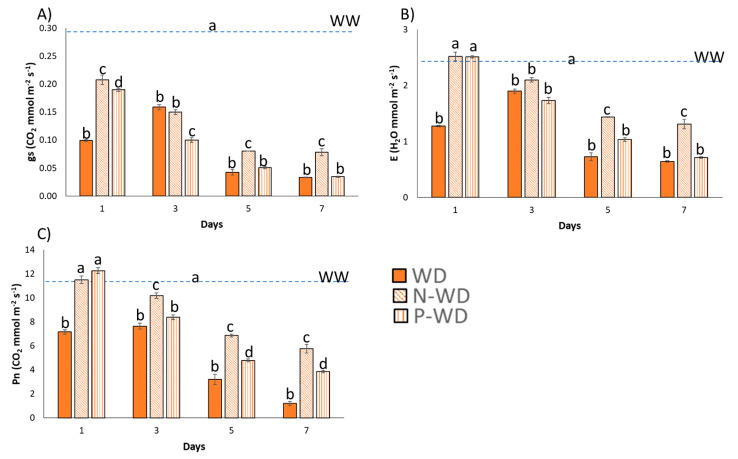
Average of (**A**) stomatal conductance, (**B**) evapotranspiration, and (**C**) net photosynthesis with standard deviation. The blue dashed line represents the values of well-watered plants (full values are displayed in the Supporting Information) (N = 20 seedlings). WD means plants subjected to water deficit; P means pyroglutamic treatment; N means Nisco^®^ microparticles treatment. Bars with the same letter show no significant differences (*p* < 0.05) using One-way ANOVA tests (Duncan’s post hoc).

Plants subjected to water deficit without treatment showed a significant reduction in stomatal conductance, evapotranspiration, and net photosynthesis (Figure 4A–C). In contrast, plants treated with encapsulated pyroglutamic acid (N-WD) exhibited higher values in these parameters, even surpassing those treated with the free amino acid formulation (P-WD).

**Figure 5:**
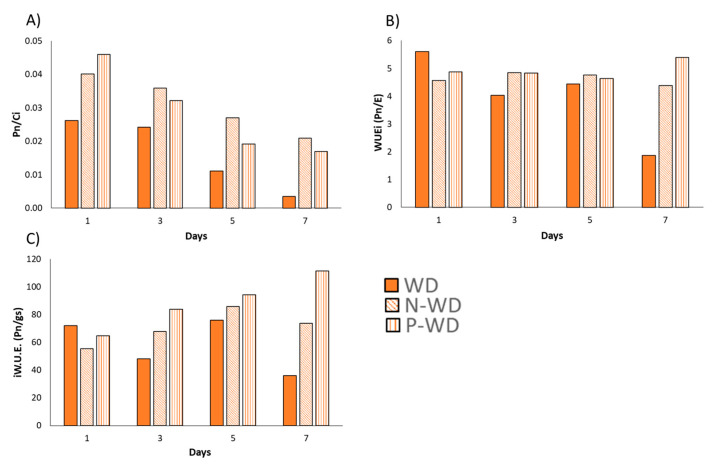
(**A**) Pn/Ci ratio, (**B**) instantaneous water use efficiency, and (**C**) intrinsic water use efficiency. WD means plants subjected to water deficit; P means pyroglutamic treatment; N means Nisco^®^ microparticles treatment.

When the data were analyzed through various ratios, the beneficial effects of pyroglutamic acid became more evident. The treated plants maintained higher Pn/Ci ratios and exhibited elevated levels of intrinsic water use efficiency (iWUE) and instantaneous water use efficiency (WUEi) three days after the onset of water deprivation. These findings highlight the biostimulant’s capacity to enhance plant tolerance under stress conditions (Figure 5A–C).

**Figure 6:**
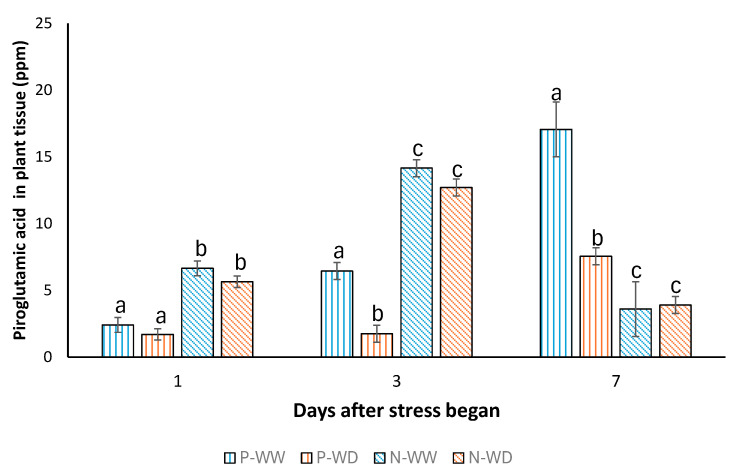
Pyroglutamic acid absorption average profile with standard deviation in tomato plants after 1 to 7 days (N = 2 seedlings). WW means well-watered plants; WD means plants subjected to water deficit; P means pyroglutamic treatment; N means Nisco^®^ microparticles treatment. Bars with the same letter show no significant differences (*p* < 0.05) using Student’s *t* test.

Finally, a pyroglutamic acid absorption profile was conducted. Figure 6 demonstrates that encapsulation significantly enhances the uptake of pyroglutamic acid within plant tissues at both 1 and 3 days post-treatment. Notably, after seven days, plants treated with P-WD exhibit a clear absorption of this amino acid, occurring four days earlier than the absorption observed in plants treated with P-WW. This indicates that certain factors limit pyroglutamic acid accumulation in plants. However, the use of alginate beads aids to increase the amino acid accumulation.

## 4. Discussion

Environmental changes are a major concern affecting field yields. Climate change is projected to reduce global crop yields by 3–12% by mid-century and by 11–25% by the end of the century [23], highlighting the necessity of developing new strategies. It is expected to exacerbate agriculture’s environmental impacts by reducing productivity, decreasing agrochemical efficacy, increasing crop pests and soil erosion, and driving further land clearing, species extinctions, and greenhouse gas emissions. In response to yield losses, farmers may intensify agrochemical use, further aggravating pollution and emissions. The combined effects of these processes—acting independently, additively, or multiplicatively—climate change as a significant amplifier of agriculture’s environmental footprint [24]. Given this daunting scenario, researchers must focus their efforts on developing innovative treatments to help producers reduce labor impacts and achieve more sustainable agricultural practices [24].

Biostimulants play a crucial role in helping farmers tackle climate challenges and increase productivity sustainably. They improve crop resilience and nutrient efficiency, which aligns directly with the European Green Deal and the Farm to Fork Strategy by promoting reduced chemical inputs and healthier soils [24] This makes biostimulants key tools for advancing sustainable agriculture and meeting ambitious environmental targets in Europe. Aligned with European policies [25] promoting increased crop yields with minimal environmental impact, pure organic active compounds offer an effective alternative for precisely managing environmental effects. In this context, pure organic active compounds present an alternative for precisely controlling environmental effects [8] as their interactions are easier to study compared to other biostimulant sources, such as extracts or microbial products. However, their use is often limited by their rapid degradation in the environment due to biotic and abiotic factors [26]. This degradation results in a short residence time in the soil, necessitating continuous applications to maintain their protective activity [5,6]. Consequently, this significantly increases costs, often rendering these treatments unaffordable for farmers.

Pyroglutamic acid exemplifies this situation perfectly. It is a natural amino acid found in plants [4] and represents a promising option to enhance crop yields under water stress [5,6], with a minimal environmental impact [6]. However, its high cost limits its widespread use by farmers [6]. This challenge led us to explore strategies to improve product dosage, focusing on protecting the active compound and ensuring its availability to plants, even under water deficit conditions. For this purpose, we employed alginate bead microencapsulation, a method that, to our knowledge, has not been previously studied for encapsulating amino acids in agriculture. Alginate offers multiple advantages: it is a cost-effective material compared to other encapsulation methods reported in the literature [27], facilitates product delivery and controlled release [28], and is easy to handle, making industrial-scale applications feasible [27].

Since preparation methodologies can influence the physicochemical properties of alginate-based composites, as well as encapsulation efficiency and release profiles [29], this study evaluated two methodologies for creating alginate beads using calcium chloride as the binding agent. The first method is the Nisco^®^ Var J1 encapsulation system, which operates on the basic principle of using a coaxial air stream to pull droplets from a needle tip into a gelling bath [30]. The second method is electrospray, a technique derived from electrospinning. Electrospinning is a physical process in which polymer fibers are drawn by the action of an electric field, without the need for additional mechanical energy [13]. Our results indicate that the particles obtained using both methodologies did not differ significantly in size (Figure 1 and Figure 2); however, the Nisco^®^ system produced droplets with a lower size dispersion. This difference may play a key role in the observed variations in encapsulation efficiency, as it reflects fundamental mechanistic distinctions between the two methods. The superior encapsulation efficiency achieved with the Nisco^®^ system can be attributed to its air-jet cutting mechanism, where a laminar jet of the alginate solution is intersected by a concentric airflow, generating droplets at regular intervals. This process results in more uniform droplets compared to those produced by electrospray, enabling more consistent bead formation. In both systems, droplets are introduced into a calcium chloride gelling bath under stirring. However, the broader dispersion of electrosprayed droplets leads to the formation of a larger number of smaller particles. Their higher surface-area-to-volume ratio increases the likelihood of biostimulant diffusion out of the droplets before complete gelation. Furthermore, electrospray is more sensitive to factors such as solution conductivity, flow rate, and ambient conditions [31], which can cause variability in droplet size and stability. In contrast, the Nisco^®^ system allows for straightforward control over droplet size and production rate, contributing to a more reproducible and efficient encapsulation process.

Given these advantages, it is hardly surprising that Nisco^®^ microparticles were more effective in increasing tolerance, even when using only half the number of particles compared to those produced by the electrospray method (Figure 3). This outcome is directly related to the fact that the encapsulation efficiency achieved with the Nisco^®^ system was more than twice that of the beads produced using the electrospray (Table 3). The Nisco^®^ VAR J1 system, employed in this study, demonstrated superior encapsulation efficiency and offers notable advantages in scalability and process control compared to electrospray. Its air-jet cutting mechanism is compatible with parallel nozzle configurations, enabling increased throughput and serving as a basis for scale-up using modular systems [30]. While electrospray faces challenges in scaling due to technical and environmental sensitivities, Nisco^®^ provides a more robust and controllable solution for producing alginate-encapsulated biostimulants. Although fully turnkey industrial-scale encapsulation systems are not yet widely available, these configurations provide a strong foundation for process development and future scale-up, including custom-engineered solutions.

As demonstrated previously, pyroglutamic acid treatment enhances tolerance to water-deficit stress in crops such as tomato [21] and maize [6] (Figure 3A,B). This study reveals that encapsulation reduces the required concentration of pyroglutamic acid by a factor of ten using both encapsulation methodologies. Remarkably, Nisco^®^ microparticles induce stress tolerance with a concentration twenty times lower than that of the free amino acid.

A detailed growth analysis revealed that the water deficit reduced the relative growth rate (RGR) of tomato plants by 50%, while biostimulant treatments—regardless of encapsulation—improved tolerance by approximately 40% (Table 4). This improvement was reflected in higher net photosynthesis levels (Figure 5C) and a higher A/Ci ratio that indicates more efficient carbon assimilation relative to internal CO_2_ concentration water-deficit conditions (Figure 5A). The presented data demonstrate that pyroglutamic-treated plants exhibited increased evapotranspiration and stomatal conductance, indicating better osmotic adjustment under drought stress [32]. This enhanced osmotic adjustment is likely due to the rapid conversion of pyroglutamic acid into glutamic acid within the plant [5]. Glutamic acid may serve as a precursor that primes the proline biosynthesis pathway, with proline acting as a well-established compatible osmolyte that aids the plant in coping with stress conditions [4]. Additionally, these plants showed improved instantaneous water use efficiency and intrinsic water use efficiency (Figure 5B,C), reflecting their capacity to optimize carbon fixation while minimizing water loss [33].

Interestingly, during the water-deficit period (Table 4), the encapsulated treatment achieved higher levels of water use efficiency (WUE) and lower levels of the stress susceptibility index (SSI). Enhanced WUE is associated with increased crop productivity under limited water supply [33], while a lower SSI indicates greater stress tolerance [34]. Following rehydration, differences in performance between WD and P-WD plants became less pronounced, while remaining consistently lower in the N-WD treatment. These findings highlight the potential of encapsulation to amplify biostimulant efficacy, even at significantly reduced concentrations. Additionally, similar improvements in water saving and yield performance have been observed through utilizing L-pyroglutamic acid in maize trials in field conditions [6], which are able to save 30% of water requirements, reaching higher yields compared with untreated crops. However, to be effective, two treatments of 500 g per hectare are required. These involve an extra cost per hectare of EUR 40 per treatment to reduce watering by 30% with some losses in yield. The use of alginate and calcium chloride to reduce the amount of biostimulant is more cost-effective due to their low price at an industrial scale. Furthermore, the additional costs associated with encapsulation should be absorbed by manufacturers, as it remains profitable for them and beneficial for farmers. The results presented in this study, demonstrating at least a tenfold reduction in concentration, are highly promising for enhancing the feasibility of application in productive systems, although they remain preliminary and require validation under field conditions. However, this raises several questions for future research, including the feasibility of utilizing industrial-grade alginate. As previously discussed, there is also a need to develop novel polymer encapsulation systems suitable for industrial-scale production.

Another noteworthy finding concerns the absorption profile of pyroglutamic acid in tomato plants (Figure 6). In plants treated with the encapsulated form, amino acid levels began to rise after just one day and peaked at three days, regardless of the irrigation level. This pattern contrasts markedly with that observed for the free amino acid treatment, where maximum accumulation occurred after seven days and was significantly affected by water limitation. This delayed response in the free amino acid treatment may be related to root growth, as the more dispersed compound in its free form takes longer to reach the roots effectively. Another interesting finding concerns the pyroglutamic acid absorption profile in tomato plants (Figure 6). Encapsulated pyroglutamic acid-treated plants began to show an increase in amino acid concentration after one day, reaching the highest concentration after three days, regardless of the amount of water used. This behavior contrasts sharply with the free amino acid treatments profile, where the maximum concentration was reached after seven days, and was significantly affected by water restriction. We attribute this difference to the localized application of microparticles near the base of the plant, where they remain on the soil surface within the rhizosphere. Upon irrigation—even with 50% less water—the amino acid is consistently released near the roots, improving its bioavailability. Alginate beads are known to exhibit a rapid release profile, with most of the compound released within 1 to 3 h under in vitro conditions. This is consistent with previous studies, which have shown that alginate microbeads typically display low encapsulation efficiency and fast release kinetics for highly water-soluble compounds, such as pyroglutamic acid.

**Figure 7:**
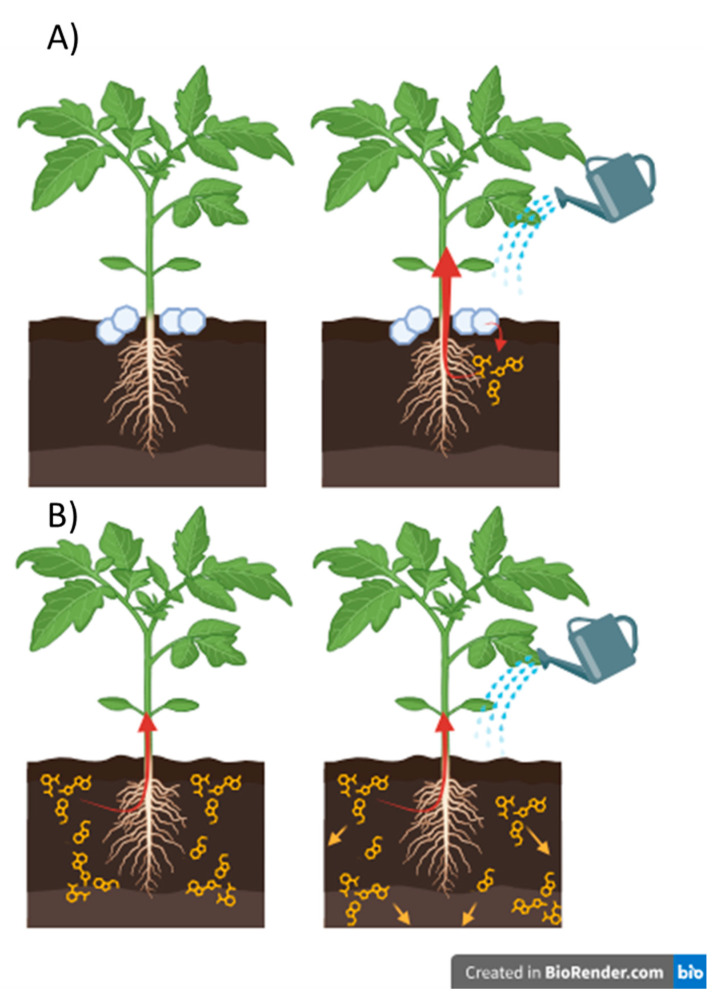
Schematic representation of the behavior of calcium alginate beads loaded with pyroglutamic acid (**A**) and the non-encapsulated treatment (**B**).

As a result, encapsulated biostimulants provide a more targeted and sustained delivery to the root zone, enhancing absorption efficiency. In contrast, the free amino acid—despite being applied at ten times the concentration—diffuses more broadly throughout the soil, reducing its availability to the roots, particularly under water-stress conditions (Figure 7). It is plausible that root growth and extension are required for effective uptake of the dispersed free amino acid, which could explain the delayed increase in amino acid concentration observed in root tissues compared to plants treated with alginate-encapsulated biostimulants (Figure 6). This result highlights a key consideration for the development of effective formulations: in vitro release profiles may not accurately reflect performance in soil environments, underscoring the importance of evaluating delivery systems under realistic application conditions.

Additionally, we examined the implications and potential applications of these findings using previous data [6] and considering the tenfold lower concentration, which demonstrates how encapsulation can enhance efficiency when using pure organic active compounds, such as pyroglutamic acid. Our results indicate that the use of alginate microcapsules can be an effective tool for improving biostimulant treatments, considering that it is an eco-friendly treatment capable of degrading in the field without posing any environmental risk [14]. Nevertheless, we would like to highlight the need for continued research in biopolymer encapsulation for agricultural applications and its advancement towards industrial-scale implementation. While biopolymers have already shown promising applications in drug transport and protection in other fields, their potential in agriculture remains relatively underexplored.

## 5. Conclusions

As climate change impacts global agriculture, there is a growing need to develop sustainable strategies that can improve crop resilience to environmental stress. Pyroglutamic acid, a naturally occurring amino acid, has been shown to have the potential to improve plant tolerance to water stress. It acts as a biostimulant that can help mitigate the adverse effects of drought, but its widespread application has been limited by its rapid degradation in the soil and its high cost. Encapsulation emerges as a solution to protect this valuable compound, allowing for a more efficient use and reducing the need for frequent applications.

- Two encapsulation methods (Nisco^®^ and electrospray) were employed, with Nisco^®^ being more effective in increasing tolerance to water deficit, even with half the amount of microparticles. This may be due to Nisco’s higher encapsulation efficiency, which reached 74.5%.
- Additionally, the Nisco^®^ particles were more homogeneous in size, contributing to a more consistent release profile and better overall performance compared to those produced by electrospray.
- Encapsulation reduced the pyroglutamic acid dosage by a factor of ten, significantly improving drought tolerance in tomato and maize crops.
- The encapsulated form also enhanced pyroglutamic acid absorption and improved parameters like photosynthesis, stomatal conductance, and water use efficiency.

The use of biopolymers for encapsulation in agricultural treatments holds great promise for sustainable crop production, particularly in the face of climate change. Exploring the potential of alternative natural polymers introduces an exciting field of research, aligning with the European Union’s “Farm to Fork” strategy. This strategy emphasizes sustainability and reduces environmental impact in food production, making biopolymer-based solutions integral for meeting these goals. However, for these solutions to be adopted on a wide scale, they must also be economically viable, ensuring that they are both sustainable and cost-effective for farmers.

## Figures and Tables

**Table 1:** Treatments and abbreviations used in the experiments.

Treatments	Conditions
WW	Well-watered untreated plants
WD	Water-deficit untreated plants
E-WD	Water-deficit plants plus 34 mg * of empty microparticles
N-WW	Well-watered plants plus 34 mg * of Nisco^®^ microparticles
N-WD	Water-deficit plants plus 34 mg * of Nisco^®^ microparticles
ES-WD	Water-deficit plants plus 34 mg * of electrospray microparticles
P-WD	Water-deficit plants 5 mL of 1 mM of pyroglutamic acid

* Dosage was optimized in a previous experiment. To deliver an amount of pyroglutamic acid equivalent to the 1 mM free treatment, approximately 340 mg of microparticles would need to be applied per plant. A tenfold dilution corresponds to 34 mg, while a twentyfold dilution corresponds to 17 mg.

**Table 2:** Stress index used in the experiments ^a^.

Index	Formula
stress susceptibility index	SSI = (1 − (Dws/Dwp)/SII
stress intensity index	SII = 1 − (Dws/Dwp)
tolerance to stress index	STI = (Dws × Dwp)/Dwp
relative growth rate	RGR = (ln Dw2 − ln Dw1)/(t2 − t1)
water use efficiency	WUEp = Dw2/water used ^b^
stress susceptibility index	SSI = (1 − (Dws/Dwp)/SII

^a^ Dws, Dwp, Dws, and Dwp represent dry weight under stress, weight under nonstress for each treatment, and weight means in stress and nonstress conditions for all treatments, respectively. Dw1 and Dw2 indicate seedling dry weights at times t1 and t2 (t1 is the beginning and t2 is the end of the period studied), respectively. Wf, Wd, and Wt refer to fresh, dry, and turgor weights, respectively. ^b^ Considering all water used over the experimental period.

**Table 3:** Particle encapsulation efficiency.

	Encapsulated Pyroglutamic Acid (ppm) ^a^	Encapsulation Average	Encapsulation Efficiency (%) ^a^
Nisco^®^ microparticles	3.35	3.3 ± 0.76 *	74.5
	3.24		
	3.38		
Electrospray microparticles	1.49	1.48 ± 0.66	33.2
	1.41		
	1.54		

^a^ Values were calculated based on the maximum possible encapsulation of pyroglutamic acid (4.46 ppm initial concentration) at 1 mM to produce 350 mg of microparticles. * Indicates significant differences between the two microparticle types at *p* < 0.05.

**Table 4:** Stress indices studied over water deficit and after rehydration.

Treatment	Water-Deficit Period (7 Days)				Rehydration Period (7 Days)		
	R.G.R ^a^	W.U.E	SSI	STI	R.G.R	SSI	STI
WW	0.2	5.8			0.25		
N-WW	0.2	5.8			0.26		
P-WW	0.2	5.8			0.26		
WD	0.11	6.3	1.3	0.5	0.22	1.1	0.8
N-WD	0.15	8.3	0.8	0.7	0.24	0.8	0.8
P-WD	0.15	7.8	0.9	0.7	0.24	1	0.8

^a^ RGR, relative growth rate; WUE, water use efficiency; SSI, stress susceptibility index; STI, stress tolerance index; WW means well-watered plants; WD means plants subjected to water deficit; P means pyroglutamic treatment; N means Nisco^®^ microparticles treatment.

## Data Availability

The data used to create the figures and tables can be found in the Supplementary Materials, except for Figure 4 and Figure 5, which will be available only under request due to their complexity.

## Supplementary Materials

The following supporting information can be downloaded at: https://www.mdpi.com/article/10.3390/polym17121617/s1, Figure S1: Nisco experimental set up and conditions; Figure S2: Electrospray experimental set up and conditions; Figure S3: UHPLC-QToF-MS chromatograms of pyroglutamic acid (PGA) in a sample analyzed. Table S1: Figure data; Table S2: Table 4 full growth dataset.
