# Supplementary figures and images for: Alginate Microencapsulation as a Tool to Improve Biostimulant Activity Against Water Deficits
Source: Polymers (Basel). 2025 Jun 10;17(12):1617. doi: 10.3390/polym17121617 (PMC12197189; doi:10.3390/polym17121617)

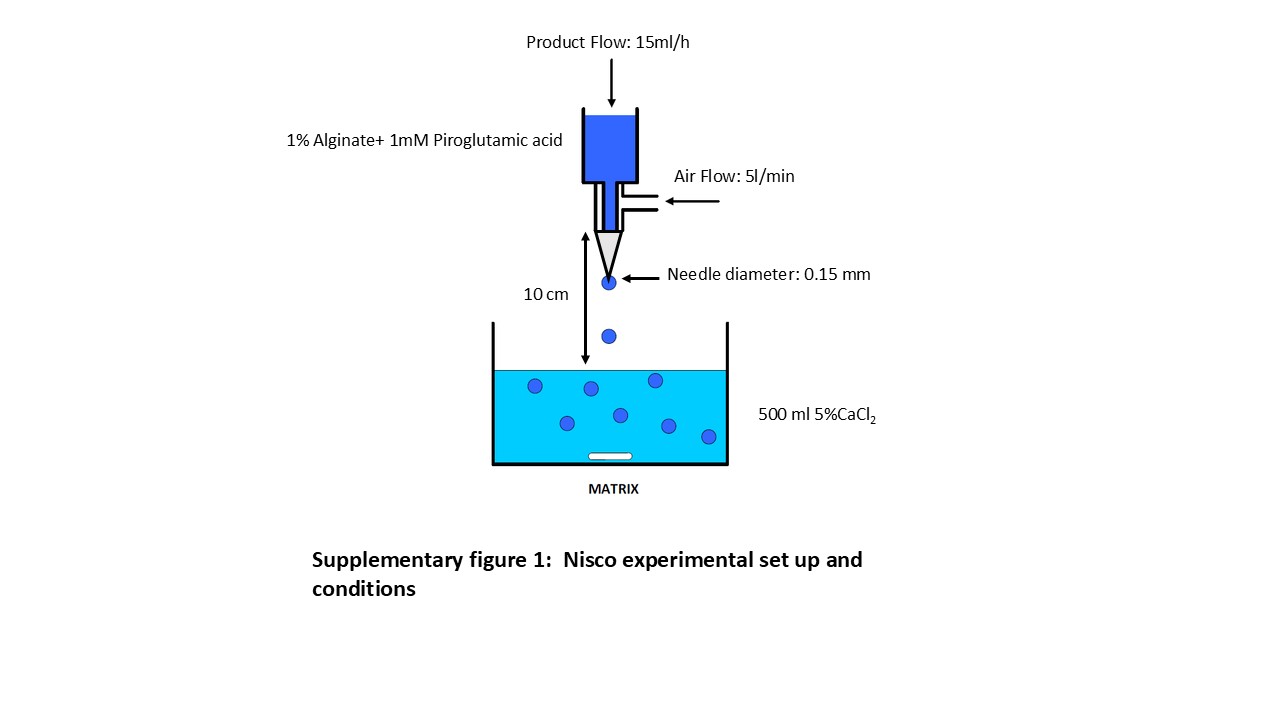

Supplement: Supplementary file 1 [file polymers-17-01617-s001.zip › Supplementary figure 1.JPG]

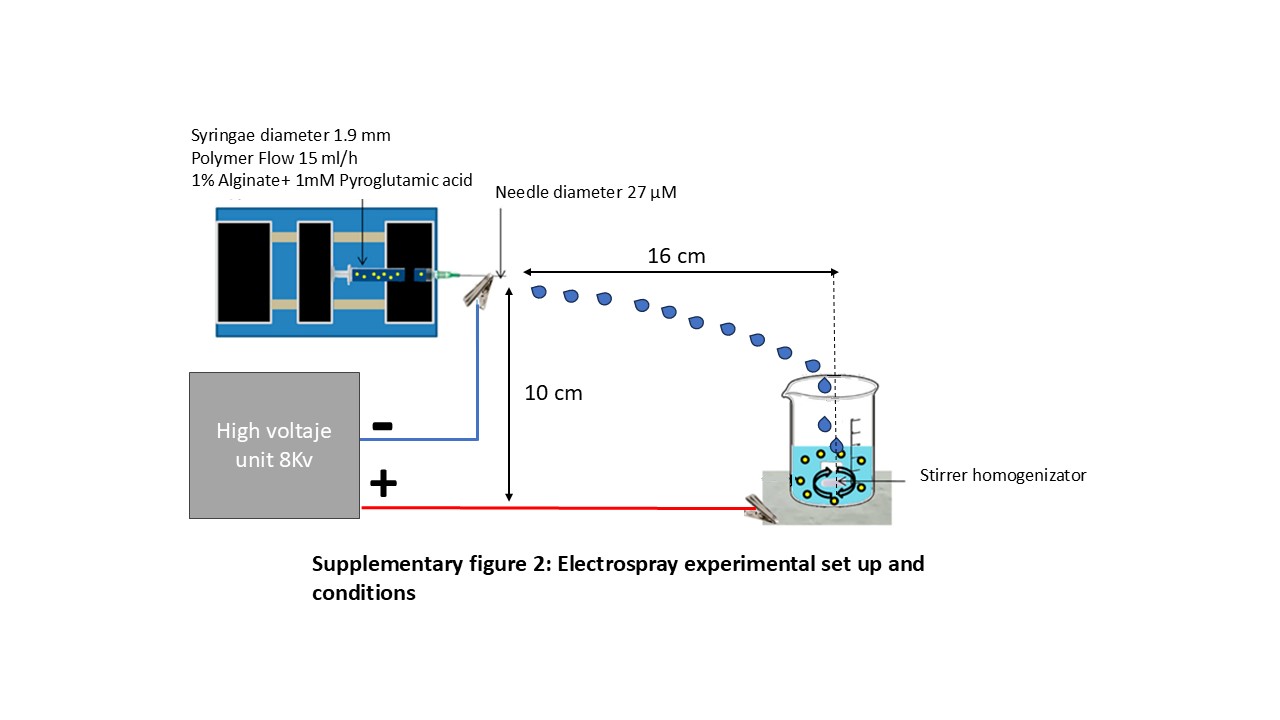

Supplement: Supplementary file 1 [file polymers-17-01617-s001.zip › Supplementary figure 2.JPG]

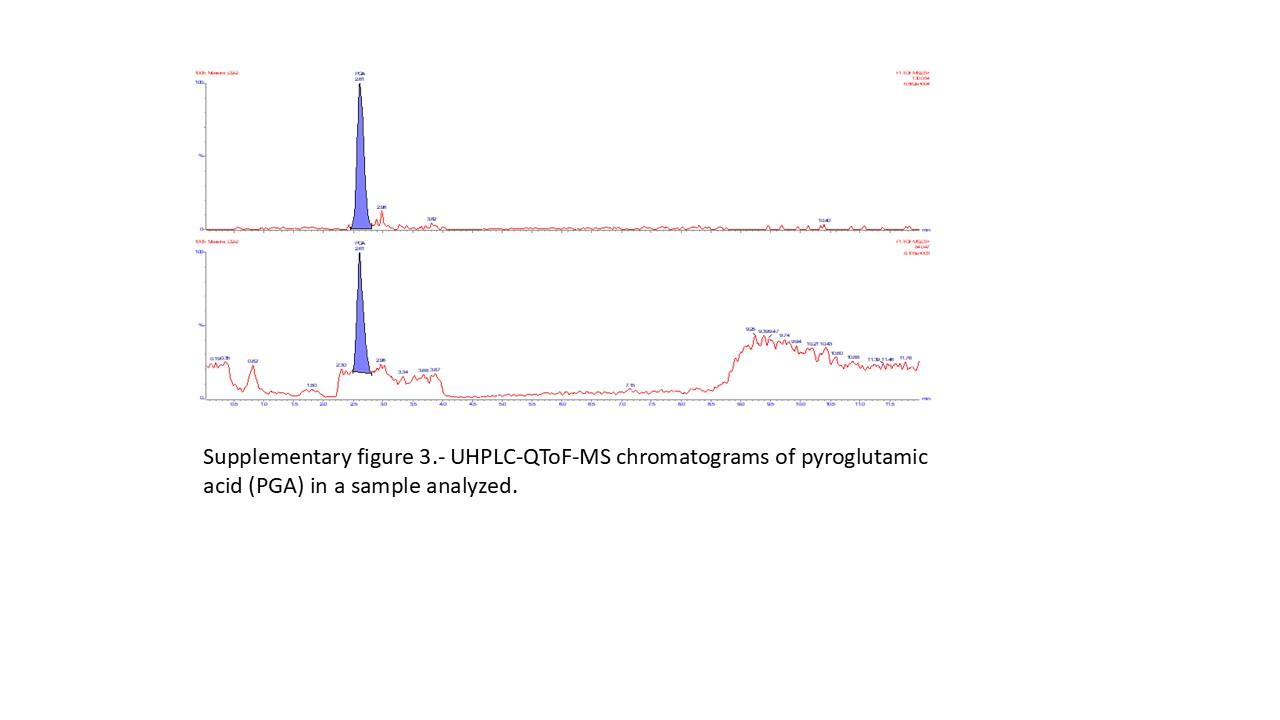

Supplement: Supplementary file 1 [file polymers-17-01617-s001.zip › Supplementary figure 3.JPG]
